# Supplementary material for: To Inject or to Reject? The Body Image Perception among Aesthetic Dermatology Patients
Source: J Clin Med. 2022 Dec 26;12(1):172. doi: 10.3390/jcm12010172 (PMC9821449; doi:10.3390/jcm12010172)
Supplement: Supplementary file 1 [file jcm-12-00172-s001.zip › jcm-1994100-supplementary.pdf]

Table S1. The influence of cosmetic procedures and treatment satisfaction history on body appreciation (FAS, BAS-2) and body image (BSQ-16) outcomes.

	First time AP (Y/N)	Treatment satisfaction after APs (Y/N)
FAS	N.S.	N.S.
BAS-2	N.S.	N.S.
BSQ-16	N.S.	N.S.

FAS – Functionality Appreciation Scale, BAS-2 – Body Appreciation Scale-2 , BSQ-16 – Body Shape Questionnaire - 16 , AP – aesthetic procedure
N.S. – not significant ($p>0.05$)
